# The French national registry for rare diseases: an integrated model from care to epidemiology and research

**DOI:** 10.1186/1750-1172-9-S1-O7

**Published:** 2014-11-11

**Authors:** Rémy Choquet, Paul Landais

**Affiliations:** 1 BNDMR, Assistance Publique Hôpitaux de Paris, Hôpital Necker Enfants Malades, Paris, France; 2 INSERM, U1142, LIMICS Paris, France; 3 EA2415, Clinical Research University Institute, Faculty of Medicine, Montpellier University & BESPIM, Nîmes University hospital, France

## 

The first national plan for rare diseases (2005-2008) set the network for care and research in the rare diseases (RD) field across all French hospitals. The 131 RD centers of expertise (CE) initiated then various IT projects to register electronically their RD patients. The CEMARA project [1] up to now registered 235,000 RD patients, from 62 RDCE (out of 131), 383 units of care and described over 4000 rare diseases. The identified limits of the CEMARA model were: i) data collection was not incorporated in the care setting, ii) exposed to data re-entry, iii) coping with data privacy new regulations and iv) gaining a wide national consensus on the data to collect for all RDs and from all CEs. To overcome these limits, the 2^nd^ national plan for rare diseases (2010-2014) promoted the creation of a national data repository for all rare diseases (BNDMR) based on the CEMARA model with the following objectives: i) identifying RD patients within the health information systems in care setting, ii) describing the RD demand of care, and the adequacy of the supply and iii) identifying patients eligible for clinical trials or cohorts.

The proposed national architecture incorporates a national minimum data set for all rare diseases (F-MDS-RD) into hospital information care systems to enable electronic patient files re-use for epidemiological studies or research [2]. To ensure a full interoperability between local hospital information systems and the BNDMR, a national interoperability framework is defined. It is set on 3 pillars: i) defining a national patient ID for rare diseases that will help to identify patients across different health IS, ii) defining a common data format for all rare diseases, compatible with EHR standards such as HL7, and iii) setting the necessary technical data flows complying with strict security rules for data privacy [3] and security.

The minimum data set for rare diseases is now being implemented in several local systems, and connectors are being rolled out with several database suppliers. Data re-entry is nowadays a major concern for clinicians. Enabling data re-use is not only an interoperability problem; data must be structured, qualified, standardized [4] and suitable for research [5]. The structuration of the data collected is set on a variety of standard terminologies such as Orphanet or the Human Phenotype Ontology which requires to be accompanied with the necessary IT tools to help clinicians coding the data [6].
